# A Portrait of the Transcriptome of the Neglected Trematode, *Fasciola gigantica*—Biological and Biotechnological Implications

**DOI:** 10.1371/journal.pntd.0001004

**Published:** 2011-02-01

**Authors:** Neil D. Young, Aaron R. Jex, Cinzia Cantacessi, Ross S. Hall, Bronwyn E. Campbell, Terence W. Spithill, Sirikachorn Tangkawattana, Prasarn Tangkawattana, Thewarach Laha, Robin B. Gasser

**Affiliations:** 1 Department of Veterinary Science, The University of Melbourne, Werribee, Australia; 2 School of Animal and Veterinary Sciences, Charles Sturt University, Wagga Wagga, Australia; 3 Department of Pathobiology, Faculty of Veterinary, Medicine, Khon Kaen University, Khon Kaen, Thailand; 4 Department of Anatomy, Faculty of Veterinary Medicine, Khon Kaen University, Khon Kaen, Thailand; 5 Department of Parasitology, Faculty of Medicine, Khon Kaen University, Khon Kaen, Thailand; University of Pittsburgh, United States

## Abstract

*Fasciola gigantica* (Digenea) is an important foodborne trematode that causes liver fluke disease (fascioliasis) in mammals, including ungulates and humans, mainly in tropical climatic zones of the world. Despite its socioeconomic impact, almost nothing is known about the molecular biology of this parasite, its interplay with its hosts, and the pathogenesis of fascioliasis. Modern genomic technologies now provide unique opportunities to rapidly tackle these exciting areas. The present study reports the first transcriptome representing the adult stage of *F. gigantica* (of bovid origin), defined using a massively parallel sequencing-coupled bioinformatic approach. From >20 million raw sequence reads, >30,000 contiguous sequences were assembled, of which most were novel. Relative levels of transcription were determined for individual molecules, which were also characterized (at the inferred amino acid level) based on homology, gene ontology, and/or pathway mapping. Comparisons of the transcriptome of *F. gigantica* with those of other trematodes, including *F. hepatica*, revealed similarities in transcription for molecules inferred to have key roles in parasite-host interactions. Overall, the present dataset should provide a solid foundation for future fundamental genomic, proteomic, and metabolomic explorations of *F. gigantica*, as well as a basis for applied outcomes such as the development of novel methods of intervention against this neglected parasite.

## Author Summary

*Fasciola gigantica* (Digenea) is a socioeconomically important liver fluke of humans and other mammals. It is the predominant cause of fascioliasis in the tropics and has a serious impact on the lives of tens of millions of people and other animals; yet, very little is known about this parasite and its relationship with its hosts at the molecular level. Here, advanced sequencing and bioinformatic technologies were employed to explore the genes transcribed in the adult stage of *F. gigantica*. From >20 million raw reads, >30,000 contiguous sequences were assembled. Relative levels of transcription were estimated; and molecules were characterized based on homology, gene ontology, and/or pathway mapping. Comparisons of the transcriptome of *F. gigantica* with those of other trematodes, including *F. hepatica*, showed similarities in transcription for molecules predicted to play roles in parasite-host interactions. The findings of the present study provide a foundation for a wide range of fundamental molecular studies of this neglected parasite, as well as research focused on developing new methods for the treatment, diagnosis, and control of fascioliasis.

## Introduction

Liver flukes are socio-economically important parasitic flatworms (Platyhelminthes: Trematoda: Digenea) affecting humans and livestock in a wide range of countries. Two key representatives are *Fasciola gigantica* and *F. hepatica*. These parasites are the main cause of fascioliasis, a significant disease in ungulates [1]–[3] and humans, which is usually contracted *via* the ingestion of contaminated aquatic plants [4]. Fascioliasis due to *F. gigantica* is recognized as a neglected tropical disease and is estimated to affect millions of people, mainly in parts of Africa, the Middle East and South-East Asia [2], [5]–[10].

*Fasciola gigantica* and *F. hepatica* share common morphological, phylogenetic and biological characteristics, most clearly inferred by the evidence of sustained *F. gigantica* x *F. hepatica* (i.e. hybrid or introgressed) populations [11]–[13]. *Fasciola* spp. have di-heteroxenous life cycles [2], [14] which involve (freshwater) lymnaeid snails as intermediate hosts and mammalian definitive hosts. The pathogenesis of fascioliasis in the definitive host is characterized by two main phases: (i) the *acute/subacute phase* begins with the ingestion of the metacercarial stage on herbage and is characterized by tissue damage, caused by the migration of immature worms through the duodenal wall, and then the liver capsule and parenchyma (usually 2–6 weeks) [1]. Clinical signs can include abdominal pain, fever, anaemia, hepatomegaly and weight loss; (ii) the *chronic phase* commences when adult worms have established in the biliary ducts (∼7–8 weeks after infection) [1]. In addition to hepatic fibrosis (following acute/subacute infection) and anaemia, the chronic phase is characterized by progressive cholangitis, hyperplasia of the duct epithelium and periductal fibrosis, which can result in cholestatic hepatitis [15], [16]. The onset of clinical signs can be variable, slow and typically include anaemia, jaundice, inappetence, oedema/ascites and/or diarrhoea [17], [18]. Fascioliasis can also sometimes be associated with complications, such as co-infections with anaerobic bacteria [1], [10].

Despite their substantial morphological and biological similarities, differences in host specificity between *F. gigantica* and *F. hepatica* appear to define the aetiology and clinical manifestation of disease in the definitive host [2]. A well-characterized difference between these parasites is their adaptation to different intermediate snail hosts. *Fasciola gigantica* usually prefers snail species (e.g., *Radix natalensis* and *R. rubiginosa*) that live in warm climates, whereas *F. hepatica* often utilizes snails (e.g., *Lymnaea tomentosa* and *Galba truncatula*) that are widespread in cool climates [19]. This difference in intermediate host-preference appears to affect the distribution of the parasites, with *F. gigantica* being the most common cause of fascioliasis in the tropics and *F. hepatica* being more common in temperate regions. In sub-tropical regions, where both species of *Fasciola* can co-exist, fascioliasis is reported to be associated with *F. gigantica*, *F. hepatica* and/or *F. gigantica* x *F. hepatica* hybrid populations [2], [19]. The clinical manifestation of fascioliasis in definitive hosts can also depend on parasite factors (e.g., species/strain of worm, infective dose and/or intensity of infection) and host factors (e.g., species of host, immune response and phase/duration of the infection) [1]–[3], [20]–[23]. Some studies seem to suggest that *F. gigantica* may be better adapted to parasitize cattle, with higher levels of resistance being observed in sheep and goats [20], [21], [24]. In contrast, most breeds of sheep are highly susceptible to fascioliasis caused by *F. hepatica* [20]. Current evidence [2], [20], [24], [25] suggests differences in biology between *F. gigantica* and *F. hepatica* as well as the disease(s) that these parasites cause; yet, our understanding of the molecular biology of these parasites and of fascioliasis, particularly in humans, is in its infancy [16], [26].

Recent developments in high-throughput sequencing [27]–[30] and bioinformatics [31] are now providing researchers with the much-needed tools to explore the fundamental biology of digeneans [32], [33]. To date, molecular biological research of socioeconomically important trematodes has been dominated by a focus on *Schistosoma mansoni* and *S. japonicum*, culminating, recently, in the sequencing of their nuclear genomes [34], [35]. These two genome sequences provide an invaluable resource to support fundamental explorations of the biology and evolution of flukes as well as their interactions with their hosts [35]. However, the biology of schistosomes, which live *en copula* (i.e. as male/female pairs) in the blood stream of mammalian hosts, is distinct from that of hermaphroditic liver flukes, such as *F. gigantica* and *F. hepatica*. Recently, the transcriptomes of several foodborne liver flukes, including *F. hepatica*, *Clonorchis sinensis* and *Opisthorchis viverrini*, were determined [36], [37]. Although this progress has improved our understanding of the molecular biology of these worms and has paved the way toward the discovery of new intervention targets, almost nothing is known about *F. gigantica*. This paucity of knowledge is clearly illustrated by the comparison of >60,000 transcripts currently available for *F. hepatica* [36], [38], [39] with a total of 39 for *F. gigantica* in public databases (National Center for Biotechnology Information, NCBI).

In the present study, we characterized the transcriptome of the adult stage of *F. gigantica* and provide an essential resource for future explorations of this socioeconomically important parasite. We used massively parallel nucleotide sequencing of a non-normalized cDNA library to provide a deep insight into this transcriptome as well as relative transcription levels in this developmental stage. In addition, comparative analyses of the dataset predicted a range of proteins that are conserved among trematodes, providing an invaluable resource to underpin future efforts toward developing new approaches for the intervention against and control of fascioliasis.

## Materials and Methods

### Collection of adult *F. gigantica*

Adults of *F. gigantica* were collected (at an abattoir in Khon Kaen, Thailand), from the large bile ducts of a liver from a water buffalo (*Bubalus bubalis*) with a naturally acquired infection. All work was conducted in accordance with protocols approved by the animal ethics committee of the Department of Anatomy, Faculty of Veterinary Medicine, Khon Kaen University, Thailand. Adult worms were washed extensively in physiological saline and then transferred to and maintained in culture *in vitro* for 2 h [36] to allow the worms to regurgitate caecal contents. Subsequently, all worms were washed extensively in physiological saline, snap-frozen in liquid nitrogen and then stored at −80°C. The specific identity of each individual worm was verified by isolating genomic DNA [40] and conducting PCR-coupled, bidirectional sequencing (ABI 3730xl DNA analyzer, Applied Biosystems, California, USA) of the second internal transcribed spacer (ITS-2) of nuclear ribosomal DNA [36]. In addition, the reproductive state and ploidy of each of three adult worms used for transcriptomic sequencing were examined histologically [41]; the presence of mature eggs and sperm confirmed that all three worms represented *F. gigantica* and not *F. gigantica* x *F. hepatica* hybrids (see [11]).

### Library construction and sequencing

A full poly(A)-selected transcriptome sequencing approach (RNA-seq) was employed. *DN*ase I-treated total RNA was extracted from three adult worms of *F. gigantica* using the TriPure isolation reagent (Roche), according to manufacturer's protocol. The amounts of total RNA were determined spectrophotometerically, and RNA integrity was verified by agarose gel electrophoresis and using a 2100 BioAnalyzer (Agilent). Polyadenylated (polyA+) RNA was purified from 10 µg of total RNA using Sera-Mag oligo(dT) beads, fragmented to a length of 100–500 nucleotides, reverse transcribed using random hexamers, end-repaired and adaptor-ligated, according to the manufacturer's protocol (Illumina). Ligated products of ∼200 base pairs (bp) were excised from agarose and PCR-amplified (15 cycles). Products were cleaned using a MinElute column (Qiagen) and sequenced on a Genome Analyzer II (Illumina), according to the manufacturers' instructions.

### Assembly and remapping of short-insert Illumina reads

The short-insert, single reads, generated from the adult *F. gigantica* cDNA library, were assembled using the computer program SOAPdenovo v1.04 [42]. Briefly, short-insert, single-end reads filtered for adapter sequences and suboptimal read quality (i.e. with PHRED quality scores of <28) were used to construct and store a De Bruijn-graph using a *k*-mer value of 29 bp. Sequence reads were trimmed, and links with low coverage were removed before contig sequence *k*-mers were conjoined in an unambiguous path. To reduce apparent redundancy, sequences of *>*200 nucleotides were clustered using the contig assembly program (CAP3) [43], employing a minimum overlap length of 40 nucleotides and an identity threshold of 95%. Using BLASTn and then BLASTx analyses, all nucleotide sequences (n = 12) with significantly higher identity (based on the *E*-value) to those of any potential contaminants (including bacteria, fungi and/or the bovid host) than to digeneans or any other eukaryotes (for which sequence data are currently available) were removed.

The raw sequence reads derived from the non-normalized adult *F. gigantica* cDNA library were then mapped to the non-redundant transcriptomic data using the program SOAP2 [44]. Briefly, raw sequence reads were aligned to the non-redundant transcriptomic data, such that each raw sequence read was uniquely mapped (i.e. to a unique transcript). Reads that mapped to more than one transcript (designated “multi-reads”) were randomly allocated to a unique transcript, such that they were recorded only once. To provide a relative assessment of transcript abundance, the number of raw reads that mapped to each sequence was normalized for length (i.e. reads per kilobase per million reads, RPKM) [45].

The non-redundant transcriptomic dataset for adult *F. gigantica* was annotated (April 2010) based on BLASTx for protein sequence homology at permissive (*E*-value: <1E^−05^), moderate (<1E^−15^) and/or stringent (<1E^−30^) search strategies against sequences in available databases, including: (i) NCBI non-redundant sequence database (GenBank, http://www.ncbi.nlm.nih.gov/); (ii) non-redundant genome-wide sequence databases for eukaryotic organisms [ENSEMBL (http://www.ensembl.org/), SchistoDB for *S. mansoni* (http://schistodb.net/schistodb20/) [46] and the Chinese Human Genome Center at Shanghai database for *S. japonicum* (http://lifecenter.sgst.cn/schistosoma) [47]; (iii) transcriptomic datasets available for *F. hepatica*, *Clonorchis sinensis* and *Opisthorchis viverrini* [36], [37]; and (iv) manually curated information resources for peptidases (MEROPS database) [48] and kinases (European Molecular Biology Laboratory kinase database, http://www.sarfari.org/kinasesarfari/).

Proteins were conceptually translated from the predicted coding domains of individual nucleotide sequences. Protein-coding sequences were classified functionally using the program InterProScan [49], employing the default search parameters. Based on their homology to conserved domains and protein families, predicted proteins of *F. gigantica* were assigned gene ontology (GO) categories and parental (i.e. level 2) terms (http://www.geneontology.org/). Inferred proteins with homologues/orthologues in other organisms were mapped to conserved biological pathways utilizing the Kyoto encyclopedia of genes and genomes (KEGG) orthology-based annotation system (KOBAS) [50]. Orthologues in KEGG (i.e. metabolic) pathways were displayed using the tool iPath2 (http://pathways.embl.de/ipath2) [51]. Signal peptides were also predicted using the program SignalP 3.0, employing both the neural network and hidden Markov models [52], and transmembrane domains using TMHMM [53], a membrane topology prediction program. Proteins inferred to be classically excreted and/or secreted from *F. gigantica*, based on the presence of a signal peptide, absence of any transmembrane domain(s) as well as sequence homology to one or more known excretory/secretory (ES) proteins listed in databases for eukaryotes [54], *F. hepatica* [39], *S. mansoni* [55] and the nematode *Brugia malayi* [56], [57] were identified and collated.

## Results

### Characterization of the transcriptome of *F. gigantica*

More than 20 million, short-insert Illumina reads were generated for the adult stage of *F. gigantica* (Table 1). Raw sequence data were deposited in the sequence read archive (SRA) database of NCBI (http://www.ncbi.nlm.nih.gov/sra) under accession number SRA024257. BLASTn searches (*E*-value: 1E^−05^) revealed that all 39 expressed sequence tags (ESTs) available in public databases for this parasite were contained within the present, assembled sequence dataset (available *via* http://gasser-research.vet.unimelb.edu.au/; contact corresponding authors); thus, only the sequence data from the present study were assembled (see Table 1). Short reads clustered into 30,525 unique sequences with a mean length of 524 nucleotides (range: 201–18,098) and with a G+C content of 46.0±4.2%. More than 25% of the raw reads were re-mapped (sequence length of ≥200 nucleotides) to the transcriptomic data, with a mean depth of coverage of 188±469 reads per sequence.

**Table 1:** Characteristics of the transcriptome of the adult stage of *Fasciola gigantica*.

*Raw sequences/clusters*	
Sequence reads before assembly	21.9 million, each 76 bp in length
Total unique sequences after assembly^a^	30,525 (524±524; 201–18,098)
Possible host or other contaminant sequences quarantined	12
GC content (%)	46.0±4.2
Illumina reads mapped to sequences^b^	5.7 million (26.20%)
Coverage (number of reads mapped to each sequence)	187.8±469.0 (3 – 17,118)

*Characterization of transcriptomic data*	
Predicted protein translations from conceptual open reading frames	30,513 (145±155; 27–5,974)
Predicted proteins with signal peptides	1,543 (5.1%)
Predicted proteins with a transmembrane domain	3,599 (1–14 domains)
Sequence homologues within non-redundant sequence databases^c^ (BLASTx, *E*-value 1e^−05^)	27,755 (91.0%)
InterPro conserved coding domains	9,326 sequences (30.6%); 3,535 Interpro terms
Gene ontology (GO) terms predicted	5,183 sequences (17.0%); 1,124 GO terms
Biological process category	3,461 sequences; 401 GO terms
Cellular component category	1,980 sequences; 147 GO terms
Molecular function category	4,564 sequences; 576 GO terms
KEGG biological pathway sequence homologues	4,466 sequences (14.6%); 1,981 KO terms; 225 pathways and 18 protein families
Cellular processes	919 sequences; 324 KO terms
Environmental information processing	738 sequences; 278 KO terms
Genetic information processing	661 sequences; 355 KO terms
Human diseases	341 sequences; 165 KO terms
Metabolism	1,259 sequences; 549 KO terms
Protein families	2,214 sequences; 998 KO terms
Unclassified	384 sequences; 128 KO terms
EMBL kinase sequence homologues	308 sequences; 221 kinase terms
MEROPS peptidase sequence homologues	304 sequences; 245 MEROPS terms
MEROPS peptidase inhibitor sequence homologues	137 sequences; 122 MEROPS terms

**a** Summarized as number of sequences (average sequence length ± standard deviation; minimum and maximum sequence lengths).

**b** Summarized as number of sequences (proportion of total sequences used for the analysis).

**c** Based on homology to sequences within NCBI non-redundant, *Clonorchis sinensis*, *Fasciola hepatica*, *Opisthorchis viverrini*, *Schistosoma mansoni* and *S. japonicum* sequence databases.

### Sequence homology between *F. gigantica* and key eukaryotes

The transcriptomic dataset was used to interrogate genomic/transcriptomic databases (i.e. *F. hepatica*, *C. sinensis*, *O. viverrini*, *S. mansoni*, *S. japonicum* and NCBI non-redundant sequence databases) using BLASTx. The majority of *F. gigantica* sequences (27,755 of 30,513 sequence matches, equating to 91.0%) matched previously identified molecules at an *E*-value threshold of 1E^−05^ (Table 1). Proteins inferred from the transcriptome of *F. gigantica* were compared with those predicted from transcriptomic data for the adult stages of *F. hepatica*, *C. sinensis* and *O. viverrini* [36], [37] and complete proteomic datasets for selected organisms, including *Saccharomyces cerevisiae* (yeast), *S. mansoni* and *S. japonicum* (trematodes), *Caenorhabditis elegans* (‘elegant worm’), *Drosophila melanogaster* (vinegar fly); *Danio rerio* (zebra fish), *Gallus gallus* (chicken), *Xenopus tropicalis* (frog); *Bos taurus* (cattle), *Homo sapiens* and *Mus musculus* (mouse) (Table 2). As expected, proteins predicted for *F. gigantica* (n = 30,513) had the highest sequence homology to *F. hepatica* using permissive (27,354 sequences; 89.6%), moderate (25,390 sequences; 83.2%) and stringent (20,798 sequences; 68.2%) search strategies. Amino acid sequences inferred for *F. gigantica* had the greatest similarity to those of other members of the class Trematoda included herein, resulting in 10,752 to 27,354 sequence matches (35.2–89.6%) or a total of 27,745 sequences matches (90.9%) at an *E-*value of 1E^−05^. In agreement with findings for other trematodes [34]–[37], proteins inferred for *F. gigantica* had a higher sequence similarity to those of mammals (30.1–30.2%) than *C. elegans* (23.8%).

**Table 2:** Protein sequence homology inferred between or among *Fasciola gigantica*, other parasitic trematodes, and selected eukaryotic organisms.

*F. gigantica* sequences (n = 30,513) homologous^a^ to those in:	Number of sequences with homology (%)		
	<1E^−05^	<1E^−15^	<1E^−30^
*Fasciola hepatica* b	27,354 (89.6)	25,390 (83.2)	20,798 (68.2)
*Schistosoma mansoni* b	13,762 (45.1)	10,135 (33.2)	6,410 (21)
*Schistosoma japonicum* b	12,913 (42.3)	9,252 (30.3)	5,543 (18.2)
*Opisthorchis viverrini* b	11,005 (36.1)	6,859 (22.5)	3,773 (12.4)
*Clonorchis sinensis* b	10,752 (35.2)	6,841 (22.4)	3,900 (12.8)
Sequences with homology to at least one member of Class Trematoda	27,745 (90.9)	25,725 (84.3)	21,100 (69.2)
*Mus musculus* c	9,222 (30.2)	5,969 (19.6)	3,323 (10.9)
*Bos taurus* c	9,226 (30.2)	5,910 (19.4)	3,262 (10.7)
*Homo sapiens* c	9,196 (30.1)	5,969 (19.6)	3,321 (10.9)
*Danio rerio* c	9,101 (29.8)	5,850 (19.2)	3,225 (10.6)
*Gallus gallus* c	8,761 (28.7)	5,596 (18.3)	3,085 (10.1)
*Xenopus tropicalis* c	8,702 (28.5)	5,563 (18.2)	3,046 (10)
*Drosophila melanogaster* c	8,267 (27.1)	5,241 (17.2)	2,854 (9.4)
*Caenorhabditis elegans* c	7,249 (23.8)	4,372 (14.3)	2,353 (7.7)
*Saccharomyces cerevisiae* c	4,175 (13.7)	2,248 (7.4)	1,175 (3.9)
Sequences with homology to at least one non-trematode	10,364 (34)	6,623 (21.7)	3,641 (11.9)

**a** All amino acid sequences conceptually translated from sequence data were searched against protein databases using BLASTx employing permissive (*E*-value of 1E^−05^), moderate (*E*-value of 1E^−15^) and stringent (*E*-value of 1E^−30^) search strategies.

**b** Sequence database contains available transcriptomic data.

**c** Sequence database contains translated proteins from entire genomic sequence data.

Comparative protein sequence analysis was carried out between or among key members of the Trematoda (Table 3). Despite significant differences in biology and life history, representatives of the family Fasciolidae (i.e. *F. gigantica* and *F. hepatica*) shared greater protein sequence homology (38.3%; *E*-value: 1E^−05^) with sequences encoded in the genomes of *S. japonicum* and *S. mansoni* (blood flukes; family Schistosomatidae) than to those encoded by transcripts from the adult stages of *C. sinensis* and *O. viverrini* (liver flukes; family Opisthorchiidae; 26.8%; *E*-value: 1E^−05^). Only a small number of proteins predicted for *F. gigantica* (i.e. 253 and 705 sequences at an *E*-value of 1E^−30^ and 1E^−05^, respectively) were homologous among the representatives of the families Fasciolidae, Schistosomatidae and Opisthorchiidae, but absent (based on a similar level of sequence homology) from the other eukaryotic organisms included in the present study (see Table S1). These molecules included proteases (mastin and leucine amino peptidase), membrane transporter proteins (aquaporin 3, multidrug resistance-associated protein-type ATP-binding cassette transporter and oxalate:formate antiporter) and proteins involved in cellular signalling (i.e. calcium binding proteins and an epidermal growth factor-like peptide).

**Table 3:** Comparative genomic analysis between or among the Trematoda^a^.

*F. gigantica* sequences (n = 30,513) homologous^b^ to those in:	Number of sequences with homology (%)		
	<1E^−05^	<1E^−15^	<1E^−30^
Fasciolidae	27,354 (89.6)	25,390 (83.2)	20,798 (68.2)
Fasciolidae and Schistosomatidae	11,678 (38.3)	8,328 (27.3)	4,940 (16.2)
Fasciolidae and Opisthorchiidae	8,179 (26.8)	4,372 (14.3)	2,120 (6.9)
Fasciolidae and Opisthorchiidae and Schistosomatidae	6,583 (21.6)	3,476 (11.4)	1,641 (5.4)
Fasciolidae and Opisthorchiidae and Schistosomatidae and ENSEMBL^c^	5,878 (19.3)	2,995 (9.8)	1,370 (4.5)
Sequences common to all trematodes and not present in ENSEMBL^c^ sequence datasets	705 (2.3)	481 (1.6)	253 (0.8)

**a** Including representatives of the Fasciolidae (*Fasciola gigantica* and *Fasciola hepatica*), Opisthorchiidae (*Clonorchis sinensis* and *Opisthorchis viverrini*) and Schistosomatidae (*Schistosoma mansoni* and *S. japonicum*).

**b** All amino acid sequences conceptually translated from sequence data were searched against protein databases using BLASTx employing permissive (*E*-value of 1E^−05^), moderate (*E*-value of 1E^−15^) and stringent (*E*-value of 1E^−30^) search strategies.

**c** Inferred proteins homologous to those of eukaryotic model organisms (cf. Table 2).

Proteins inferred from the transcriptome of *F. gigantica* were predicted to contain signal peptide domains (1,543 sequences) and/or transmembrane domains (3,599 sequences) (Table 1). Based on the presence of signal peptide domains in and absence of transmembrane motifs from the predicted proteins as well as the presence of one or more homologues in current ES protein databases, 255 putative ES proteins, including cysteine proteases, cathepsins B and L, legumain and cystatin (a cysteine protease inhibitor) were inferred (Table S2).

Predicted proteins were also categorized according to their inferred molecular function, cellular localization and association with biological pathways, and compared with those encoded in the transcriptomes of the adult stages of other liver flukes, including *F. hepatica* (Table 1 and Table S3). A significant proportion (30.6%) of the transcriptome of *F. gigantica* was inferred to encode 3,535 conserved protein domains or family signatures. Based on this annotation, 1,124 GO terms were inferred. The transcriptome of *F. gigantica* contained most of the parental (i.e. level 2) terms assigned previously to *F. hepatica* (87%) [36], *C. sinensis* and *O. viverrini* (80%) [37], based on analyses of sequence data generated previously from normalized cDNA libraries representing adult worms. Predicted proteins assigned to the term ‘biological process’ (3,461 sequences; 401 GO terms) were associated predominantly with: (i) cellular processes (3,322 sequences; 64.1%), such as protein amino acid phosphorylation and transmembrane transport; (ii) metabolic processes (2,686 sequences; 51.8%), such as protein amino acid phosphorylation and translation; and (iii) localization (863 sequences; 16.7%), such as the directed movement of substances within or between cells including the transport of solutes across a membrane. Proteins assigned to the term ‘molecular function’ were mainly linked to: (i) binding (3,362 sequences; 70.1%), such as the binding of ATP, zinc ion and protein; (ii) catalytic activities (2,736 sequences; 52.8%) of enzymes, including protein kinases; and (iii) transporter activity (342; 6.6%), including ATPase activity, coupled to the transport of molecules through membranes. Predicted proteins for *F. gigantica* were also linked to cellular components, such as membranes, nucleus, protein complexes or ribosomes (Table S3).

Significant similarity (*E*-value: 1E^−05^) between protein sequences predicted for *F. gigantica* and those in the KOBAS database allowed 4,466 sequences to be assigned to 1,981 KO terms and 225 standardized KEGG pathway terms (Table 1). A significant proportion of amino acid sequences were associated with: (i) metabolic pathways (1,259 sequences; 549 KO terms), including carbohydrate, amino acid and lipid metabolism; (ii) cellular processes (919 sequences; 324 KO terms), including those linked to cell communication as well as the endocrine and/or immune systems; (iii) environmental information-processing pathways (738 sequences; 278 KO terms), including signal transduction, membrane transport and signaling molecules; (iv) genetic information processing pathways (661 sequences; 355 KO terms), including folding, sorting and degradation, translation and replication and repair; and (v) pathways linked to human diseases (341 sequences; 165 KO terms), including cancers, neurodegenerative disorders and infectious diseases (Table 4). Inferred proteins of *F. gigantica* (2097 sequences; 892 KO terms) were mapped to conserved, orthologous KEGG metabolic pathway terms, with a high degree of confidence based on protein sequence homology, employing moderate (785 KO terms; 88.0%; *E*-value, 1E^−15^) and stringent (589 KO terms, 66.0%; *E*-value, 1E^−30^) search strategies (Figure S1). Proteins predicted for *F. gigantica* that shared highest homology to conserved metabolic enzymes of eukaryotes (listed in the KEGG database) were associated predominantly with carbohydrate, lipid and/or energy metabolism. A high degree of similarity in metabolic pathways was evident between *F. gigantica* and *F. hepatica* [36] (Figure S2), regardless of whether the data were derived from a non-normalized cDNA library sequenced by Illumina (*F. gigantica*) [present study] or a normalized library sequenced using 454 technology (*F. hepatica*) [36]. Interestingly, in *F. gigantica*, there was no evidence of any transcripts encoding 3-oxoacyl-[acyl-carrier-protein] synthase II [EC:2.3.1.179], which, in eukaryotes, is usually linked to the fatty acid biosynthesis pathway (KEGG pathway map00061). Although this molecule was encoded in *F. hepatica* (Figure S2) and *S. mansoni* [34], it is the only enzyme representing this particular pathway in these organisms. Current evidence (cf. [58], [59]) indicates that digeneans lack the repertoire of enzymes required for the *de novo* synthesis of fatty acids and that they are highly dependent on complex fatty acid precursors from their host(s).

**Table 4:** Biological pathways inferred from the transcriptome of the adult stage of *Fasciola gigantica*.

Parent KEGG pathway^a^	Sequences^b^	Top KEGG pathway terms^c^
*Cellular processes*		
Behavior	1 (0.0)	Circadian rhythm [ko04710]
Cell communication	510 (11.4)	Focal adhesion [ko04510]; Tight junction [ko04530]
Cell growth and death	274 (6.1)	Cell cycle [ko04110]; Cell cycle - yeast [ko04111]
Cell motility	151 (3.4)	Regulation of actin cytoskeleton [ko04810]
Development	92 (2.1)	Axon guidance [ko04360]; Dorso-ventral axis formation [ko04320]
Endocrine system	481 (10.8)	Insulin signaling pathway [ko04910]; Melanogenesis [ko04916]
Immune system	290 (6.5)	Antigen processing and presentation [ko04612]; Leukocyte transendothelial migration [ko04670]
Nervous system	144 (3.2)	Long-term potentiation [ko04720]; Long-term depression [ko04730]
Sensory system	42 (0.9)	Olfactory transduction [ko04740]; Taste transduction [ko04742]
*Environmental information processing*		
Membrane transport	154 (3.4)	ABC transporters [ko02010]; Other ion-coupled transporters [ko00000]
Signal transduction	798 (17.9)	MAPK signaling pathway [ko04010]; Calcium signaling pathway [ko04020]
Signaling molecules and interaction	97 (2.2)	ECM-receptor interaction [ko04512]; Cell adhesion molecules (CAMs) [ko04514]
*Genetic information processing*		
Folding, sorting and degradation	278 (6.2)	Ubiquitin mediated proteolysis [ko04120]; Proteasome [ko03050]
Replication and repair	97 (2.2)	Other replication, recombination and repair proteins [ko00000]; DNA polymerase [ko03030]
Transcription	83 (1.9)	RNA polymerase [ko03020]; Basal transcription factors [ko03022]
Translation	207 (4.6)	Ribosome [ko03010]; Aminoacyl-tRNA biosynthesis [ko00970]
*Human diseases*		
Cancers	535 (12.0)	Colorectal cancer [ko05210]; Prostate cancer [ko05215]
Infectious diseases	47 (1.1)	Epithelial cell signaling in *Helicobacter pylori* infection [ko05120]
Metabolic disorders	36 (0.8)	Type II diabetes mellitus [ko04930]; Type I diabetes mellitus [ko04940]
Neurodegenerative disorders	105 (2.4)	Huntington's disease [ko05040]; Alzheimer's disease [ko05010]
*Metabolism*		
Amino acid metabolism	486 (10.9)	Lysine degradation [ko00310]; Glutamate metabolism [ko00251]
Biosynthesis of polyketides and nonribosomal peptides	8 (0.2)	Biosynthesis of vancomycin group antibiotics [ko01055]; Polyketide sugar unit biosynthesis [ko00523]
Biosynthesis of secondary metabolites	75 (1.7)	Limonene and pinene degradation [ko00903]; Alkaloid biosynthesis II [ko00960]
Carbohydrate metabolism	630 (14.1)	Starch and sucrose metabolism [ko00500]; Glycolysis/Gluconeogenesis [ko00010]
Energy metabolism	221 (4.9)	Oxidative phosphorylation [ko00190]; Carbon fixation [ko00710]
Glycan biosynthesis and metabolism	182 (4.1)	N-Glycan biosynthesis [ko00510]; O-Glycan biosynthesis [ko00512]
Lipid metabolism	324 (7.3)	Glycerophospholipid metabolism [ko00564]; Glycerolipid metabolism [ko00561]
Metabolism of cofactors and vitamins	191 (4.3)	Folate biosynthesis [ko00790]; Nicotinate and nicotinamide metabolism [ko00760]
Metabolism of other amino acids	96 (2.1)	Selenoamino acid metabolism [ko00450]; Glutathione metabolism [ko00480]
Nucleotide metabolism	253 (5.7)	Purine metabolism [ko00230]; Pyrimidine metabolism [ko00240]
Xenobiotics biodegradation and metabolism	138 (3.1)	Benzoate degradation via CoA ligation [ko00632]; Caprolactam degradation [ko00930]

**a** Pathway mapping was based on homology to annotated proteins in the Kyoto encyclopedia of genes and genomes (KEGG) database.

**b** Within parentheses is the percentage of the total number of sequences predicted to be homologous to KEGG orthologues.

**c** The two most frequently reported KEGG pathways.

Most abundantly transcribed genes (as assessed based on RPKM) in adult *F. gigantica* were those linked to reproductive processes, antioxidant molecules (thioredoxin, peroxiredoxin and fatty acid-binding proteins), molecular chaperones (heat shock proteins 70 and 90), proteins involved in the glycolytic pathway (fructose-bisphosphate aldolase, fructose-16-bisphosphatase-related protein, glutamate dehydrogenase and glyceraldehyde phosphate dehydrogenase), translation (elongation factor-1 alpha, RNA-binding protein 9 and cytosolic 80S ribosomal protein L39), cytoskeletal proteins (alpha-tubulin and dynein) and cysteine (calpain, cathepsin B, legumain-1 and legumain-2) and metallo (prolyl carboxypeptidase) proteases (Table S4). A detailed examination of the data revealed that a full complement of proteins required to degrade carbohydrates to phosphoenolpyruvate *via* the glycolytic pathway [58] was present (Figure S3).

**Figure 1:**
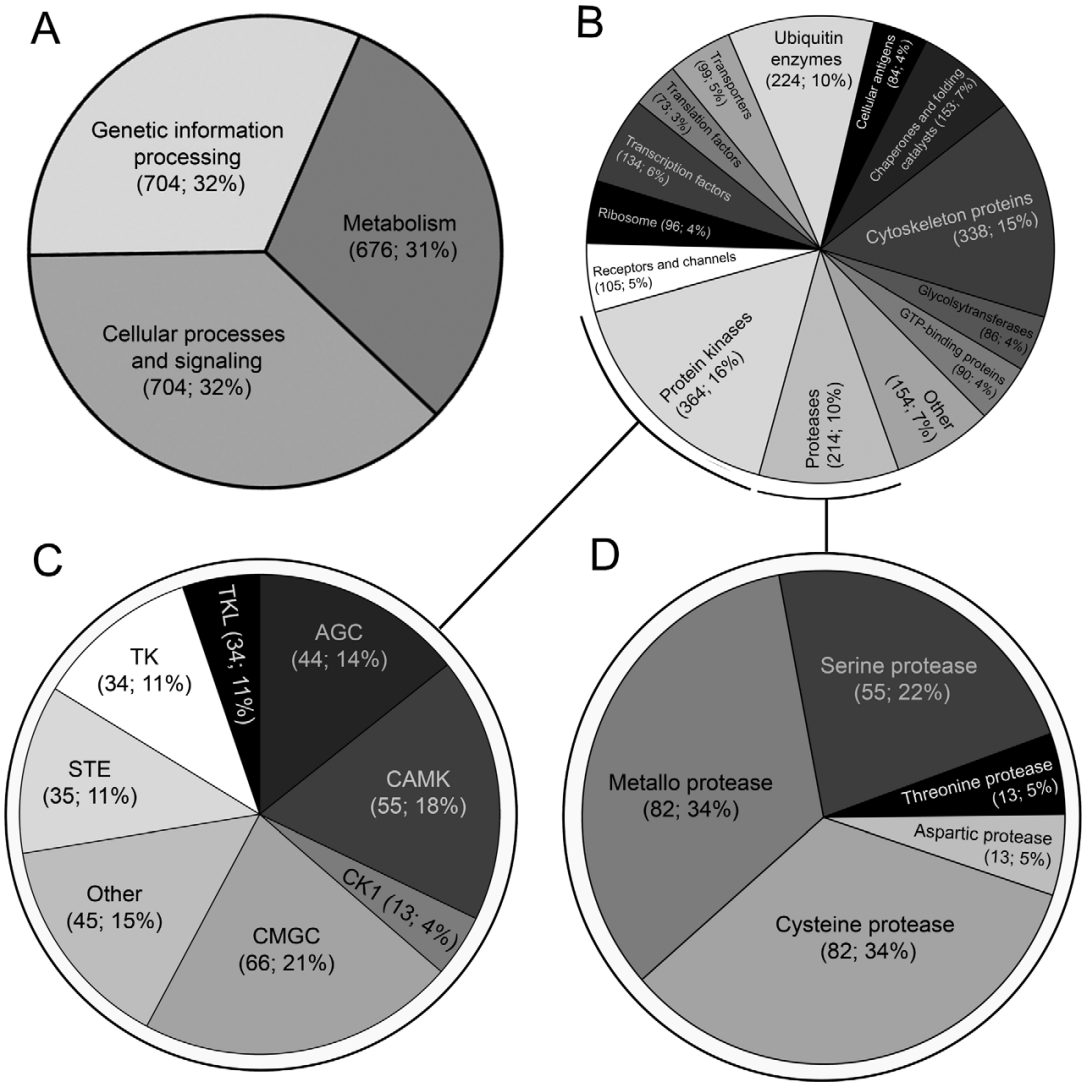
A summary of conserved groups of protein inferred from the transcriptome of the adult stage of *Fasciola gigantica*. Absolute numbers and percentages are given in parentheses. Category 1 (A) and category 2 (B) proteins were inferred based on homology to proteins in the Kyoto encyclopedia of genes and genomes (KEGG) database. Protein kinases (C) and proteases (D) were inferred based on homology to proteins in the EMBL Sarfari kinase and/or MEROPS databases. Kinase-like molecules were grouped within the: cyclin-dependent, mitogen-activated protein, glycogen synthase and CDK-like serine/threonine kinases (CMGC); Ca2+/calmodulin-dependent serine/threonine kinases (CAMK); cAMP-dependent, cGMP-dependent and protein kinase C serine/threonine kinases (AGC); serine/threonine protein kinases associated with the mitogen-activated protein kinase cascade (STE); tyrosine kinase (TK); tyrosine kinase-like (TKL); and other unclassified kinases (Other).

Proteins predicted for *F. gigantica* were assigned to major families (2,214 sequences; 998 terms) based on homology to annotated proteins in the KEGG protein family database. Sequences encoded in the transcriptome were almost equally subdivided into three major categories: ‘genetic information processing’ (704 sequences; 31.8%), ‘cellular signaling’ (704 sequences; 31.8%) and ‘metabolism’ (676 sequences; 30.5%) (Figure 1A). Putative proteins were further categorized into various sub-categories, including: (i) protein kinases (364 sequences; 16.4%); (ii) cytoskeleton proteins (338 sequences; 15.3%); (iii) ubiquitin enzymes (224 sequences; 10.1%); and (iv) proteases (214 sequences; 9.7%) (Figure 1B). A further *in silico* analysis assigned most of the protein kinases (308 sequences) to eight structurally-related classes, inferred to be crucial for normal cellular processes (Figure 1C and Table S5), such as: (i) CMGC (66 sequences; 21.4%) cyclin-dependent (CDKs), mitogen-activated protein (MAP kinases), glycogen synthase (GSK) and CDK-like serine/threonine kinases; (ii) CAMK (55 sequences; 17.9%), Ca^2+^/calmodulin-dependent serine/threonine kinases; (iii) AGC (44 sequences; 14.3%), cAMP-dependent, cGMP-dependent and protein kinase C serine/threonine kinases; (iv) STE (35 sequences; 11.4%), serine/threonine protein kinases associated with the mitogen-activated protein kinase cascade; and (v) tyrosine kinases (34 sequences; 11.0%). Kinases that were abundantly transcribed included cAMP-dependent protein kinases (AGC) and casein kinases (CK1), essential for cell signal transduction; Ca^2+^/calmodulin-dependent serine/threonine kinases (CAMK) involved in calcium signalling [60]; and dual-specificity tyrosine-(Y)-phosphorylation regulated kinase 2 (CMGC) involved in the regulation of cellular growth and/or development [61].

Similarly, further of the *F. gigantica* dataset inferred 304 proteases (linked to 247 MEROPS terms) and 137 protease inhibitors (122 MEROPS terms), including representatives of five of the seven protease catalytic types defined within the MEROPS database [48] (Figure 1D and Table S6). The ratio (aspartic:cysteine:metallo:serine:threonine) of catalytic types of proteases represented in the MEROPS database [48] and present in the of transcriptome of *F. gigantica* was 5:34:34:22:5, which was comparable with those inferred from the genomes of *S. japonicum* (4:32:35:21:8) and *S. mansoni* (6:29:35:23:7) [34], [35]. In *F. gigantica*, genes encoding the metalloproteases (82 MEROPS terms; 33.5%), leucyl aminopeptidases, cytosolic exopeptidases, which cleave N-terminal residues from proteins and peptides, were abundantly transcribed. Cysteine proteases (82 MEROPS terms; 33.5%) inferred included those involved in the digestion of host proteins (legumain/asparaginyl endopeptidase and cathepsins) and calcium-induced modulation of cellular processes (calpain) (Table S4). Like all eukaryotes, *F. gigantica* was inferred to possess a rich diversity of serine proteases (55 MEROPS terms; 22.4%), including an abundantly transcribed serine carboxypeptidase, which are presumably important for fundamental cellular processes. Threonine proteases (13 MEROPS terms; 5.3%) which were abundantly represented included enzymes required for the assembly and activation of the proteasome complex [62]. Aspartic proteases encoded (13 MEROPS terms; 5.3%) included cathepsin D, an aspartyl lysosomal peptidase which, in trematodes, is suggested to play a role in the degradation of host tissues [63].

Cathepsins representing families B and L were inferred (Table S7) from the present dataset by annotating and re-mapping sequences of ≥200 nucleotides (‘stringent conditions’). Inspection of the annotated data identified 18 and two sequences with homology to cathepsin B (including clades B1 and B2) and cathepsin L (clades 1 and 2), respectively. As cathepsin L is reported to be a dominant family of proteins of *F. gigantica* and *F. hepatica* [39], [64]–[67], the relative levels of transcription of genes encoding members of cathepsins B and L were explored. The re-mapping of raw sequences (Illumina) to previously published transcripts (n = 15) encoding cathepsins from *F. gigantica* (see Table S8) [66]–[68] revealed high (RPKM of 2,543–214,634) and low (RPKMs of 14–21) levels of transcription for 10 and two representatives, respectively, of 12 distinct members of the cathepsin L family, and low and moderate (RPKMs of 0.9 and 300) levels for two of the three representatives of cathepsin B, respectively (Table S8).

## Discussion

A number of trematodes are of major socioeconomic importance; yet, they cause some of the most neglected diseases of humans and livestock worldwide. Until recently, there has been a reliance on data and information available for schistosomes (blood flukes) [34], [35] to infer aspects of the molecular biology of key trematodes. The recent characterization of the transcriptomes of the liver flukes *F. hepatica*, *C. sinensis* and *O. viverrini* [36], [37] has provided the first insights into the molecular biology of these foodborne trematodes. Extending this work, the present study provides a deep exploration of the transcriptome of the adult stage of *F. gigantica*. With only 39 transcripts previously available in public databases, the *>*30,000 sequences characterized here are novel for this species and constitute a significant contribution to current databases [36], [37], [69]–[73] and an invaluable resource to advance our understanding of the fundamental biology of *F. gigantica*, its interplay with its hosts and the disease that this parasite causes. Importantly, the present transcriptomic data set will also be an essential resource for the future assembly of the nuclear genome of *F. gigantica*, assisting in the determination of gene structures, prediction of alternative transcript splicing and the characterization of regulatory elements.

The present transcriptomic dataset should, in the future, assist significantly in identifying genes linked specifically to parasitism and also to our understanding of the evolution of trematodes [74]. Based on current similarity searches, 80% (BLASTx, *E*-value 1E^−15^) to 90% (BLASTx, *E*-value 1E^−05^) of the predicted protein sequences of *F. gigantica* and *F. hepatica* were inferred to be homologues, reflecting their close biological and phylogenetic relationships [75]. More broadly, 253 protein sequences inferred for *F. gigantica* were homologous (BLASTx, *E*-value <1E^−30^) to proteins identified in other trematodes but divergent from those predicted for a range of other eukaryotes, including human, mouse, cattle, zebrafish, vinegar fly, ‘elegant worm’ and/or yeast. Although there is a paucity of data on the function of the majority of such molecules, their characterization could lead to the discovery of new targets for the design of safe trematocidal drugs and/or vaccines.

Massively parallel nucleotide sequencing from a non-normalized cDNA library and the subsequent assembly of sequence data have produced a high quality draft of the transcriptome of adult *F. gigantica* and provided invaluable insights into the relative abundance of transcripts. The assignment of molecules encoded in the transcriptome to molecular functions and biological pathways has revealed a substantial diversity of terms, comparable with those predicted for other liver flukes, including *F. hepatica* [36], *C. sinensis* and *O. viverrini* [37], and the blood fluke *S. mansoni* (http://amigo.geneontology.org/; http://schistodb.net/schistodb20/). Proteins known to be expressed in adult *F. hepatica* [39], [76], [77] were compared with those inferred from the transcriptome of *F. gigantica*. Molecules well represented in the adult transcriptomes of both *F. gigantica* [the present study] and *F. hepatica* [39] included antioxidants, heat shock proteins and cysteine proteases. Antioxidants have been suggested to play a role in host immune modulation and shown to be highly expressed throughout the life history of *F. hepatica* [39], including peroxiredoxin, thioredoxin and glutathione transferases, whose expression has been suggested to protect fasciolids from harmful, host-derived reactive oxygen species [78]–[80]. A similar protective role has also been reported for protein chaperones, such as heat shock protein-70, which have been inferred to play an important role in relation to protein folding and whose expression is proposed to be induced by one or more host immune responses to *F. gigantica* or *F. hepatica* [81]. Therefore, within the definitive host, adult stages of *F. gigantica* and *F. hepatica* appear to express repertoires of molecules that are directed toward the protection of cellular processes from the host response to liver fluke infection, including the protection from reactive oxygen species (ROS) [82]. Protection from damage caused by ROS is important, since juveniles of *F. gigantica* are susceptible (*in vitro*) to antibody-dependent cell-mediated cytotoxicity involving ROS [83].

A diverse array of proteases were abundantly represented in the transcriptome of the adult stage of *F. gigantica*, as expected based on previous proteomic studies [63], [84]. Cysteine proteases constituted a significant proportion of catalytic enzymes encoded in this species (Figure 1; Table S6), which appears to reflect their crucial roles in parasite feeding and/or immuno-modulation in the definitive host [85], [86]. A cathepsin B-like molecule (B1) was also well represented in the present transcriptome (Table S7 and Table S8). Evidence of abundant transcription of one or more homologues in the tegument and/or digestive and reproductive tracts [68] and their absence from ES products [39], [64], [66], [87] suggests one or more key functions for cathepsin Bs within the tissues of this parasite. A detailed analysis also revealed that transcripts encoding cathepsin Ls (including members of clades 1, 2 and 5; [66]) were abundant in the present dataset (Table S8), consistent with their dominance in ES products from adult *F. hepatica* [39], [66], [88].

The complexity of the cathepsins and the close relatedness of some of them were reflected in a technical challenge in the assembly of (short-read) Illumina sequence data. The abundance of many related and, apparently, paralogous and/or alternatively spliced transcripts encoding cathepsin Ls (cf.[66]) prevents accurate assemblies from short transcripts, even under stringent conditions (as used herein). This point emphasizes a limitation of the *de novo*-assembly of single-end sequences produced using short-read sequencing platforms, such as Illumina [27] and SOLiD [30], in the absence of a reference genome sequence. This limitation should be overcome in the future through the combined assembly and annotation of paired-end sequence data with medium to long sequences (e.g., of 350–1000 nucleotides) produced using alternative sequencing technology, such as 454 (Roche) [89]. Such an integrated sequencing approach, preferably in conjunction with proteomic analyses, could be used to quantitatively study transcription/expression profiles in key developmental stages and distinct phenotypes (or hybrids) of *F. gigantica* [11], [13], [90]. Although the transcriptome of the adult stage of *F. gigantica* has been defined here, there is no information on differential transcription among miracidial, sporocyst, redial, cercarial, juvenile and adult stages of this parasite. Clearly, exploring transcription among and also within all developmental stages of this parasite will have important implications for understanding development, reproduction, parasite-host interactions as well as fascioliasis at the biochemical, immunological, molecular and pathophysiological levels. Detailed knowledge of the transcriptome of *F. gigantica* will also assist in the study of developmental processes and metabolic pathways through functional genomics. Gene perturbation assays are available for *S. mansoni* and *F. hepatica* [91]–[95], suggesting that they could be adapted to *F. gigantica* for functional genomic explorations. The integration of data from comparative and functional analyses could pave the way for the development of new intervention methods against *F. gigantica*, built on the identification and of essential genes or gene products linked to key biological or biochemical pathways. For instance, phosphofructokinase (a glycolytic enzyme) is a known metabolic “choke-point” in *S. mansoni* [96], because trivalent, organic antimony compounds can inhibit worm growth *in vitro* [97]. The genes encoding phosphofructokinase and other key enzymes in the glycolysis pathway were abundantly transcribed in adult *F. gigantica* (Figure S3). Also a thioredoxin-glutathione reductase (a multifunctional detoxifying enzyme) might represent a novel drug target in *F. gigantica*, because a gene encoding a homologue of this enzyme in *S. mansoni* has been shown to be essential for life, based on functional genomic analyses [98]–[100]. Clearly, future structural and functional explorations of molecules (including kinases, proteases and their inhibitors, neuropeptides and selected structural proteins), which are recognized to be conserved among fasciolids and schistosomes and/or predicted to be essential and druggable [34], [101]–[104], should assist in the design and development of entirely new classes of potent trematocidal compounds.

## Supporting Information

Figure S1 A summary of metabolic pathways predicted for amino acid sequences inferred from transcriptomic data for the adult stage of *Fasciola gigantica*. Mapping was conducted based on homology to annotated proteins in the Kyoto encyclopedia of genes and genomes (KEGG) pathways database. Results were displayed using iPath2 (http://pathways.embl.de/ipath2/). The colours represent sequence homology (BLASTx) to orthologous proteins at permissive (yellow path; *E*-value <1E^−05^), moderate (orange; *E*-value <1E^−15^) and stringent (red; *E*-value, <1E^−30^) search strategies.
(TIF)

Figure S2 A summary of metabolic pathways predicted for amino acid sequences inferred from the transcriptome of the adult stage of *Fasciola gigantica* and *Fasciola hepatica* [36] based on homology mapping to annotated proteins in the Kyoto encyclopedia of genes and genomes (KEGG) biological pathways database. Results were displayed using iPath2 (http://pathways.embl.de/ipath2/). Shared pathways (green) between *F. gigantica* (yellow) and *F. hepatica* (blue) are indicated.
(TIF)

Figure S3 The glycolysis pathway predicted for proteins inferred to be encoded in the transcriptome of the adult stage of *Fasciola gigantica* based on homology mapping to annotated proteins in the Kyoto encyclopedia of genes and genomes (KEGG) biological pathways database. Levels of transcription are inferred from sequencing depth and are represented by the number of reads per kilobase per million reads (RPKM). Transcription was ranked as high (red, RPKM *>*500), moderate (orange, RPKM 250–500) or low (yellow, RPKM <250). The present image was modified from that in the KEGG database (http://www.genome.jp/kegg/).
(TIF)

Table S1 Adult *Fasciola gigantica* transcripts homologous to predicted proteins from other trematodes.
(XLSX)

Table S2 Putative adult *Fasciola gigantica* proteins with homology to classically secreted/excreted molecules.
(XLSX)

Table S3 Predicted function of adult *Fasciola gigantica* transcripts based on gene ontology (GO).
(XLSX)

Table S4 Putative adult *Fasciola gigantica* proteins with homology to proteins submitted to the NCBI non-redundant database.
(XLSX)

Table S5 Putative adult *Fasciola gigantica* proteins with homology to kinases within the European Molecular Biology Laboratory kinase database.
(XLSX)

Table S6 Putative adult *Fasciola gigantica* proteins with homology to proteases and protease inhibitors within the MEROPS enzyme database.
(XLSX)

Table S7 Putative adult *Fasciola gigantica* proteins with homology to the cathepsin family of cysteine proteases.
(XLSX)

Table S8 Raw sequence reads generated from adult Fasciola gigantica re-mapped to known F. gigantica cathepsins.
(XLSX)
