# Supplementary figures and images for: A Portrait of the Transcriptome of the Neglected Trematode, Fasciola gigantica—Biological and Biotechnological Implications
Source: PLoS Negl Trop Dis. 2011 Feb 1;5(2):e1004. doi: 10.1371/journal.pntd.0001004 (PMC3051338; doi:10.1371/journal.pntd.0001004)

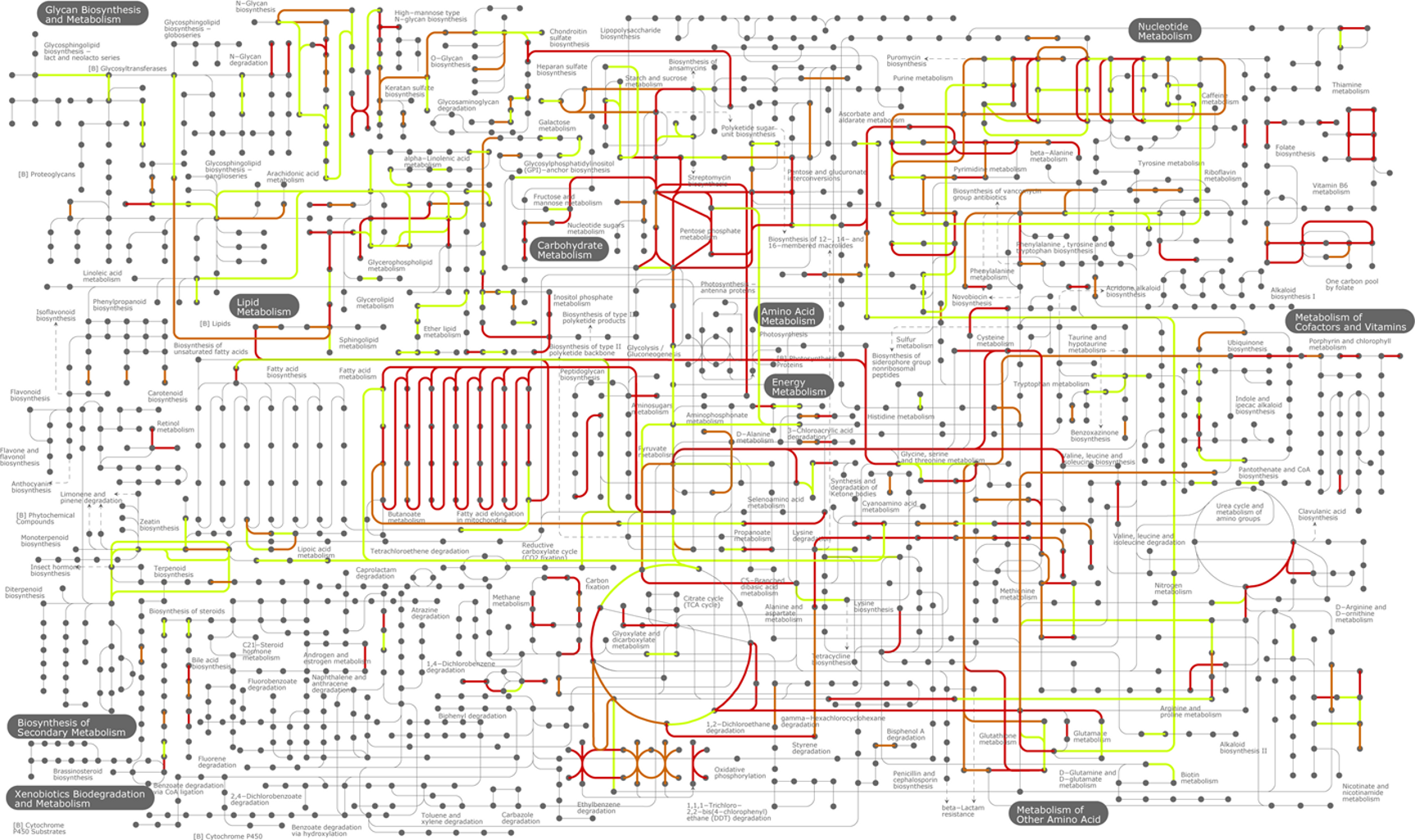

Supplement: Figure S1 — A summary of metabolic pathways predicted for amino acid sequences inferred from transcriptomic data for the adult stage of Fasciola gigantica. Mapping was conducted based on homology to annotated proteins in the Kyoto encyclopedia of genes and genomes (KEGG) pathways database. Results were displayed using iPath2 (http://pathways.embl.de/ipath2/). The colours represent sequence homology (BLASTx) to orthologous proteins at permissive (yellow path; E-value <1E−05), moderate (orange; E-value <1E−15) and stringent (red; E-value, <1E−30) search strategies. (TIF) [file pntd.0001004.s001.tif]

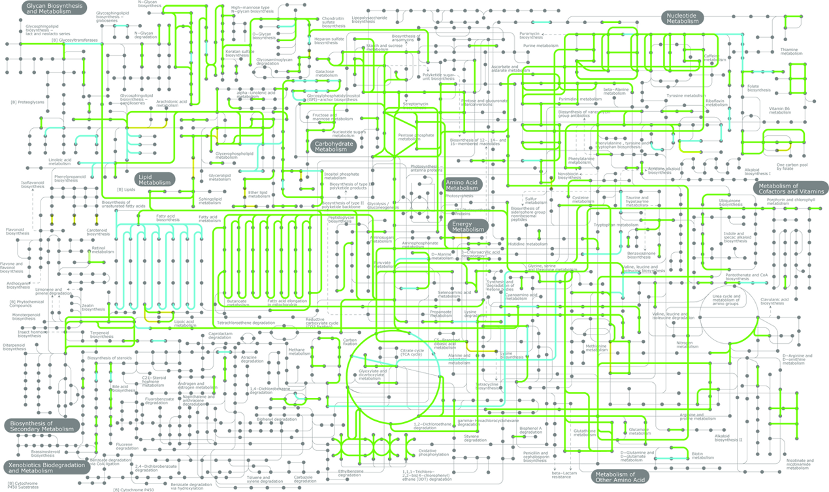

Supplement: Figure S2 — A summary of metabolic pathways predicted for amino acid sequences inferred from the transcriptome of the adult stage of Fasciola gigantica and Fasciola hepatica [36] based on homology mapping to annotated proteins in the Kyoto encyclopedia of genes and genomes (KEGG) biological pathways database. Results were displayed using iPath2 (http://pathways.embl.de/ipath2/). Shared pathways (green) between F. gigantica (yellow) and F. hepatica (blue) are indicated. (TIF) [file pntd.0001004.s002.tif]

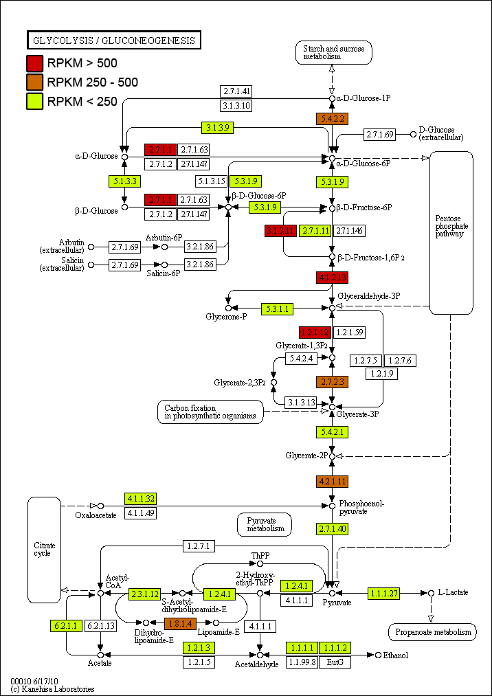

Supplement: Figure S3 — The glycolysis pathway predicted for proteins inferred to be encoded in the transcriptome of the adult stage of Fasciola gigantica based on homology mapping to annotated proteins in the Kyoto encyclopedia of genes and genomes (KEGG) biological pathways database. Levels of transcription are inferred from sequencing depth and are represented by the number of reads per kilobase per million reads (RPKM). Transcription was ranked as high (red, RPKM >500), moderate (orange, RPKM 250–500) or low (yellow, RPKM <250). The present image was modified from that in the KEGG database (http://www.genome.jp/kegg/). (TIF) [file pntd.0001004.s003.tif]
